# The Assessment of Practical and Safety Aspects of Recommended Surface Disinfection and Ultraviolet-C Cleaning Procedures for the Anesthesia Workspace

**DOI:** 10.7759/cureus.93348

**Published:** 2025-09-27

**Authors:** Andy Rho, Avery G Loftus, Franklin Dexter, Amanda Ambrose, Christopher Koo, Dan Diedrich, Breethaa J Selvamani, James Chen, Michelle C Parra, Brendan T Wanta, Jonathan E Charnin, Randy W Loftus

**Affiliations:** 1 Anesthesiology and Perioperative Medicine, University of Minnesota, Minneapolis, USA; 2 Anesthesia, University of Iowa, Iowa City, USA; 3 Anesthesiology and Perioperative Medicine, Mayo Clinic, Rochester, USA

**Keywords:** anesthesia machine, efficacy, pathogen, practicality, safety, staphylococcus aureus, surface disinfection, time, transmission, ultraviolet-c irradiation

## Abstract

Background

Cleaning procedures for the anesthesia workspace can generate substantial reductions in *Staphylococcus aureus* (*S. aureus*) transmission and all-cause surgical site infections (SSIs), including for the anesthesia machine. In this study, we evaluated their practicality and safety.

Methods

A total of seven volunteer anesthesiologists were briefly trained (two-minute video) regarding the implementation of surface disinfection and triangular (one emitter at the head and on each side of the operating room table) ultraviolet-C (UV-C) cleaning procedures. Volunteers were each randomized to three cleaning trials involving surface disinfection cleaning or surface disinfection cleaning plus two minutes of triangular UV-C treatment. The total time and efficacy of each cleaning procedure were measured along with the risk for inadvertent UV-C and ozone exposure.

Results

The mean (standard deviation (SD)) surface disinfection wiping time was 2.1 (1.5) minutes. The mean (SD) surface disinfection wiping efficacy among the seven participants was 1.13 (0.15), where 1 = some and 2 = full cleaning. The mean (SD) marginal increase for UV-C augmentation of surface disinfection wiping was 10.5 (2.1) minutes. The mean (SD) for UV-C dose delivery was 73.25 (21.85) mJ/cm^2^ for the anesthesia machine and 32.76 (15.55) for the anesthesia cart, with delivery ≥ 27.01 (0.15) mJ/cm^2^. There was zero ozone generation or inadvertent UV-C exposure.

Conclusions

Evidence-based anesthesia machine surface disinfection and triangular UV-C cleaning procedures are practical. Two minutes of triangular UV-C is a reasonable approach for the augmentation of surface disinfection that was largely ineffective, even despite training. These study results support a negligible risk for inadvertent UV-C or ozone exposure.

## Introduction

Anesthesia practitioners can generate substantial reductions in surgical site infections (SSIs) [1] that affect up to 11% of healthy patients [2] and are associated with increased mortality [3], hospital duration [4], readmission [5], and healthcare expenditure [6]. This can be achieved by improved perioperative cleaning procedures [7], including those focused on the anesthesia machine, the most potent transmission vehicle in the operating room (OR) [8].

Improved frequency and quality of cleaning of the anesthesia machine following patient induction of anesthesia/sedation and stabilization can reduce the magnitude of environmental contamination. The proportion of measured sites meeting 100 colony-forming units (CFU) per surface area sampled was reduced from 46% to 12% [9]. The reported reduction has clinical relevance, as recent work has shown that environmental sites returning ≥ 100 CFU are at greater risk of major bacterial pathogen detection in both the OR [10] and in the intensive care unit (ICU) [11]. While cleaning of the anesthesia machine with surface disinfection wipes can be efficacious [12], it can also present challenges for anesthesia practitioners. In one study, resident physicians assigned to simulated cleaning scenarios only attempted to clean five of eight sites and achieved only a 12.8%-50.0% median reduction in contamination of sites with frequent cleaning attempts, the manual bag arm/hose, and anesthesia machine tray surface. The time required for setting up the room for a planned case and cleaning is up to 10 minutes [13]. Together, prior work suggests that while important, surface disinfection cleaning of the anesthesia machine may benefit from training and augmentation with other cleaning approaches.

Ultraviolet-C (UV-C) is a reasonable consideration. Two minutes of environmental treatment with a triangular UV-C configuration, one emitter at the head of the surgical bed and one emitter on each side of the bed, can augment surface disinfection cleaning and attenuate more pathogenic bacterial strain characteristics such as *Staphylococcus aureus* (*S. aureus*) multilocus sequence type 5 (MLST5) [14] that has greater strength of biofilm formation and desiccation tolerance [15]. The minimally effective dose for achieving a six-log reduction in *S. aureus* MLST5 was a mean (standard deviation (SD)) of 27.01 (0.15) mJ/cm^2^ [14]. The impact on *S. aureus*, the sentinel pathogen for monitoring in the anesthesia workspace [16], is critically important because *S. aureus* transmission among anesthesia workspace reservoirs, including from the anesthesia machine, is tightly associated with SSI development [17]. In a subsequent study, triangular UV-C delivery was found to be noninferior to surface disinfection wipes regarding attenuation of *S. aureus* MLST5 [18].

While previously studied surface disinfection cleaning and UV-C treatment procedures have been shown to be efficacious [10-14], practical and safety considerations have not yet been addressed. Insight into these considerations may help to facilitate more widespread procedural implementation. In addition, the impact of training for surface disinfection, as suggested by prior work [13], or for UV-C implementation, has not been assessed.

In this study, our primary objectives were to assess the time required for each cleaning procedure following a brief training period, the efficacy of surface disinfection cleaning and UV-C delivery, and inadvertent UV-C exposure and ozone generation safety considerations.

## Materials and methods

Design

This was a simulation study conducted over a weekend that involved an OR and seven volunteer anesthesiologists who were recruited electronically from multiple divisions (e.g., neuro anesthesia, multispecialty, orthopedic, gynecological-oncological, and critical care). An email including a brief study description was used for recruitment.

Anesthesiologists were chosen as the study group because historical and current leaders in patient safety [19] should engage in the further development of important patient safety measures [1,7]. Further, they are a physician group that extends from prior work involving resident physicians [13], and they are particularly without prior training in cleaning procedures. The latter allowed for an assessment of the impact of the cleaning procedures following a brief training period, as previously suggested [13].

Training

Surface Disinfection Wiping

An instructional video previously uploaded by the Anesthesia Patient Safety Foundation (APSF) during the acute COVID-19 period to guide cleaning procedures (https://www.youtube.com/watch?v=ApvAdtWqs28) was displayed (approximately two minutes) for the volunteers. The video included a demonstration of surface disinfection of high-touch environmental sites, including the adjustable pressure-limiting valve and agent dial of the anesthesia machine, the Ambu bag and components of the attachment arm, the anesthesia machine tray and monitor, and the top of the anesthesia cart [13].

Triangular UV-C

Instructions for triangular positioning of the emitters (one at the head of the bed and one on each side of the bed, and within 9 ft of the anesthesia machine and equipment [20] were provided along with instructions for programming the emitters to deliver two minutes of treatment [14].

Treatment Assignment

Each of the seven volunteers was instructed to complete three cleaning trials following training. Volunteers were randomized for each trial to postinduction surface disinfection cleaning using wipes (PDI Health Care, Woodcliff Lake, NJ) alone or to surface disinfection with the addition of two minutes of triangular UV-C (Surfacide, Helios, Waukesha, WI) treatment. The randomization procedure for each trial involved randomly drawing a treatment assignment from a bin that included equal numbers of each treatment group.

Treatment efficacy measurements

Surface Disinfection Wiping

Invisible Glo Germ (Glo Germ, Moab, UT) marks were placed at environmental sites representing those cleaned in the video, including the adjustable pressure-limiting valve and agent dial of the anesthesia machine, the top of the anesthesia machine bag attachment, the anesthesia machine bag itself, the anesthesia machine tray, the bottom left-hand corner of the monitor screen, and the top of the anesthesia cart. The top of the cart was simulated via the use of a Mayo stand, as an anesthesia cart, as explained to the volunteers ahead, was not available in the simulated OR. A mark was also placed on the side of the Mayo stand (anesthesia cart) to serve as a measurement for the impact of training, as cleaning of the side of the anesthesia cart was not included in the training video. Volunteers were blinded to the placement of the invisible marks. Triangular UV-C: Calibrated radiometers (International Light Technologies model ILT1270, Peabody, MA) were placed on the anesthesia work tray by the adjustable pressure-limiting valve and dial of the anesthesia machine, and on the anesthesia cart to measure the delivered UV-C dose, which cannot be measured by Glo Germ. A delivered irradiation dose of 27.01 (0.15) mJ/cm^2^ was considered efficacious, given the proven attenuation of more pathogenic *S. aureus* strain characteristics [14].

Safety Assessments

Calibrated radiometers were placed behind the operating door window to assess the potential for inadvertent UV-C exposure through the glass window in the OR door. Supplemental oxygen on the anesthesia machine was set at 6 L/minute, and an ozone detector (Vislone, Tangxia, China) was placed in the room before the first trial and retrieved following the last trial to assess for the potential of inadvertent UV-C exposure through the glass window in the OR door.

Study outcomes

Measured outcomes included total cleaning time (the time required for UV-C treatment and surface disinfection), total UV-C (equipment retrieval, setup, treatment, and return) and surface disinfection treatment times, the efficacy of surface disinfection cleaning (0 = no cleaning (mark shape unchanged), 1 = partial cleaning (mark shape smeared, 2 = complete cleaning (mark absent)), the efficacy of UV-C dose delivery (≥ 27.01 (0.15) mJ/cm^2^), ozone detection signaled by an alarm, and UV-C irradiation detected behind the OR door.

Simple descriptive statistics were used for the analysis.

## Results

Each volunteer (N = 7) used surface disinfection wipes to attempt to clean for three trials, 21 total trials. The mean (SD) surface disinfection wiping time was 2.1 (1.5) minutes. The mean (SD) surface disinfection wiping efficacy among the seven participants was 1.13 (0.15), where 1 = some and 2 = full cleaning. The time taken to complete the wiping protocol decreased with experience, from a mean (SD) of 0.4 (0.7) minutes between the first and the third trials. Attempted cleaning occurred for 100% of 147 opportunities for measured environmental sites included in the instructional video (seven volunteers × seven measured sites × three trials = 147 total sites) and for 4.76% (1/21) of opportunities involving the side of the anesthesia cart that was not included in the instructional video (seven volunteers × one site × three trials = 21 opportunities) (Table 1).

**Table 1:** Trials, time, and the efficacy of surface disinfection and triangular ultraviolet-C (UV-C) ^#^Each of seven volunteers participated in three trials involving cleaning of seven measured locations that were reviewed in an instructional video, a total of 147 locations, seven volunteers × seven measured sites × three trials = 147 measured sites assessed. There was an attempted cleaning for 100% of the 147 measured sites. There was one site not included in the instructional video: the side of the anesthesia cart (Mayo stand). This site was cleaned for 4.76% (1/21) of opportunities (seven volunteers × one site × three trials = 21 opportunities). ^*^Total time included UV-C emitter retrieval, emitter placement, treatment, and emitter return. The actual treatment time for irradiation dose delivery was two minutes. ^@^Simulated via use of a Mayo stand. ^^^The minimally effective dose for attenuation of *Staphylococcus aureus* (*S. aureus*) is a mean (SD) of 27.01 (0.15) mJ/cm^2^ [14], where the anesthesia machine is a potent transmission vehicle [8] and *S. aureus* is the sentinel pathogen for monitoring of pathogen transmission between anesthesia workspace reservoirs [16,17,21-23]. The delivered dose exceeded the minimal threshold for 100% of treatments.

Treatment Group	
Surface Disinfection Wiping	
Number of Trials per Volunteer Mean (SD)^#^	3 (0)
Cleaning Minutes Mean (Standard Deviation)	2.1 (1.5)
Cleaning Efficacy (1 = Some, 2 = Full)	1.13 (0.15)
Decrease in Minutes First to Third Trials	0.4 (0.7)
Surface Disinfection and Triangular Ultraviolet-C (UV-C)	
Number of Trials per Volunteer Mean (SD)	1.8 (0.4)
Mean (SD) Marginal Increase in Total Minutes With UV-C^*^	10.5 (2.1)
UV-C Irradiation mJ/cm^2^ Delivered^^^	
Anesthesia Machine	73.25 (21.85)
Anesthesia Cart^@^	32.76 (15.55)

Six of the volunteers were randomized to one or more UV-C treatment simulations. Each performed a mean (SD) of 1.8 (0.4) trials. The mean (SD) marginal increase in total treatment time for augmentation of surface disinfection wiping with UV-C was 10.5 (2.1) minutes. The mean (SD) for UV-C dose delivery was 73.25 (21.85) mJ/cm^2^ for the anesthesia machine and 32.76 (15.55) for the anesthesia cart. The delivered irradiation dose exceeded 27.01 ± 0.15 mJ/cm^2^ for all measured sites and for 100% of the trials (Table 1).

There were zero alarms for ozone detection in the room or for UV-C detection outside of the OR for the entire study period.

## Discussion

The efficacy for increased frequency and quality of cleaning of the anesthesia machine through post-induction/sedation surface disinfection wiping [1,7,9] and triangular UV-C delivery [1,14,20] has been established. Practical and safety implementation considerations have not been assessed. We show in this study that surface disinfection wiping and UV-C treatment are practical and safe cleaning procedures.

Prior work suggested that anesthesia practitioners may benefit from training regarding surface disinfection, wiping, and cleaning procedures. Resident physicians without training specific to cleaning of the anesthesia machine only attempted cleaning for five of eight sites, and when they attempted cleaning, there was at most 50% efficacy [13]. In this study, previously untrained anesthesiologists received brief training with an instructional video, and there was an attempt to clean all seven measured sites in 100% of the cleaning trials. These results suggest that training was impactful, further supported by the finding that there was one attempt by one provider to clean the side of the anesthesia cart that was not included in the video, and by the decrease in cleaning time with successive trials.

The mean time for surface disinfection cleaning in this study was approximately two minutes. Prior work evaluated total OR setup and cleaning time without specifically evaluating the time required for the cleaning component [13]. While two minutes of cleaning time is practical, surface disinfection cleaning efficacy was again largely ineffective in this study, with volunteers achieving a mean score of 1.13 (0.15), where two would indicate complete cleaning (the mark eliminated). Thus, while training may increase surface disinfection wiping attempts, the persistently low efficacy of the approach indeed warrants augmentation as recommended by previously published work [14].

Environmental treatment with two minutes of triangular UV-C delivery can generate six-log reductions in *S. aureus* MLST5 for surfaces oriented vertically and horizontally to the emitters, at various heights from the floor, involving a variety of materials (e.g., textured plastic, polycarbonate, and stainless steel), and at a distance of 9 ft. The minimally effective dose required for *S. aureus* MLST5 was a mean (SD) of 27.01 (0.15) mJ/cm^2^ [14]. The triangular UV-C delivery configuration was also shown to be more effective for attenuation of *S. aureus* (ATCC 6538) and *Candida auris* (*C. auris*) vs. a single emitter rotating 360°, representing an alternative technology. The minimally effective dose for *C. auris* was 596.62 (27.98) mJ/cm^2^ [20]. The triangular configuration was also shown to be more effective for attenuation of *Clostridioides difficile* (*C. difficile*) vs. the single emitter rotating 360°, where the minimally effective dose for *C. difficile* was 432.28 (2.12) mJ/cm^2^ [21]. In this study, we show that two minutes of triangular UV-C delivered 73.25 (21.85) mJ/cm^2^ for the anesthesia machine and 32.76 (15.55) for the anesthesia cart, both positioned within 9 ft of the emitters, for seven of the seven measured sites, and for 100% of the trials. Thus, a dose sufficient to attenuate *S. aureus* [14,20] was reliably achieved when emitters were set up by first-time users. This is an important finding, as *S. aureus* transmission, including from the anesthesia machine, is tightly associated with SSI development [17], and it is the sentinel pathogen for monitoring to provide feedback to optimize preventive measures [16]. The treatment time would need to be increased accordingly for *C. auris* and *C. difficile* pathogens as previously described [20,21]. However, additional work is indicated to determine whether those pathogens are relevant considerations for the anesthesia workspace. While the dose and treatment times for Gram-negative pathogen spp. with the triangular configuration has not yet been established, Gram-negative pathogens are rarely isolated from the anesthesia machine [16], and they are not involved in the transmission of antibiotic resistance between ORs on different surgical dates, longitudinal transmission [22]. *S. aureus* has been linked by whole genome analysis to such transmission [23]. 

Triangular UV-C treatment required an average of 10 minutes of total treatment time that included equipment retrieval, setup, the two-minute treatment period, and equipment return. This total time is similar to the previously reported time for room setup and surface disinfection cleaning [13]. The actual treatment time for dose delivery was only two minutes, similar to the mean time for surface disinfection in this study. Importantly, surface disinfection with the wipes employed in this study containing the recommended alcohol and quaternary ammonium compounds [1,7] requires a drying time of three minutes for multidrug-resistant pathogens [24]. UV-C treatment is complete after two minutes. UV-C, having previously been shown to be non-inferior to surface disinfection [18], thereby offers the potential advantages of enhanced efficacy and treatment time.

Unlike surface disinfection procedures, the process for the incorporation of UV-C into intraoperative patient care has not been fully established. Relevant UV-C treatment periods include terminal cleaning at the end of the day and routine, between-case cleaning. The process of systematic implementation of UV-C to augment terminal cleaning has been studied, where one issue is to determine the number of machines required for room disinfection. To determine the number of machines required to disinfect all ORs, prior work has established that each institution should conduct a trial of nine or 19 nights to derive the number of rooms that can be treated ± two overnight [25]. Obtaining the determined number of machines is then a capital decision. Capital costs can be reduced by targeting operating/specialty combinations with the greatest numbers of infections. This methodology, using de-identified data, has been worked out precisely [26,27]. Another issue is determining treatment times that relate to personnel. This relies on understanding the target pathogen (monitoring of transmission) [1,7], the required minimally effective dose [14,20,21], limitations of the equipment (triangular vs. single emitter) [20.21], and mathematics [28].

This prior work [14,20,21,25-28] can also be leveraged to guide application of the studied technology to augment terminal cleaning for the first case of the day using four simple steps as follows.

Step 1: The OR is set up for anesthesia, including UV-C emitters obtained from storage and other anesthesia equipment (ideally, in the same location as other anesthesia equipment). The emitters would be placed at the head and on each side of the bed, and the anesthesia work area would be set up according to usual practice. With a limited supply of emitters, triangular UV-C treatment would involve OR/specialty combinations with the greatest numbers of infections (e.g., orthopedic total joint and spine surgery). This process would be executed using de-identified data with guidance using the previously described approach [25]. Prior work would be done ahead of time based on published findings to determine the number of emitters that would be required, given the target group [23].

Step 2: Before opening the surgical equipment and setting up medication infusions, all practitioners would leave the room, followed by treatment for two minutes with triangular UV-C [14] to complete cleaning before patient entry.

Step 3: Practitioners return to the room, the nursing team continues usual operations, and the anesthesia team (anesthesia practitioner, anesthesia technologist) places the emitters near the OR entry for their return to the storage location by support personnel.

Step 4: The patient enters the room, induction of anesthesia/sedation and stabilization occur, and the anesthesia machine is disinfected using a top-down approach with surface disinfection wipes containing both an alcohol and quaternary ammonium compound [1,7,9]. 

The application of UV-C to augment routine, between-case cleaning has not yet been studied. However, one can rely on clinical experience to ascertain a reasonable approach. To reduce total UV-C treatment time, the three emitters could be positioned during room setup, two minutes of triangular UV-C delivered before patient entry, and the equipment subsequently placed against the wall near OR entry for return by support personnel. This approach mirrors the use of other capital equipment, such as video laryngoscopes and ultrasound machines, that are used during routine anesthesia care. Following this approach, the net treatment time would be two minutes, and cleaning would be completed before opening the equipment, patient entry, and before anesthesia induction/sedation procedures. Future studies should formally evaluate this approach for integration into clinical practice, where the cost of two minutes of time and limited required provider activity seemingly far outweighs the improved patient safety [1,7].

We show that inadvertent UV-C exposure and ozone generation are highly unlikely scenarios in this study.

This study is limited by the simulated design. Future study/innovation should assess the real-world efficacy of the recommended approach in a randomized, controlled clinical trial involving a larger sample size and more ORs. Outcomes should focus on implementation coordination and anesthesia work area bacterial transmission dynamics. A larger study should also be conducted to solidify an approach for the augmentation of routine cleaning procedures with two minutes of triangular UV-C to address *S. aureus* pathogens and include consideration for *C. auris* and *C. difficile* pathogens, or other emerging pathogens, that may require longer treatment periods. While we studied only one UV-C technology, our prior work has shown the benefit of the triangular approach vs. alternative configurations involving a single emitter [20,21]. Future work should compare directly the dose delivery achieved by the equipment studied with other commercially available products at a clinically relevant distance of 9 ft and involving objects with horizontal orientation to the emitter(s) [20,21].

## Conclusions

Cleaning of the anesthesia machine is an important component of a multifaceted approach for improved infection control in the anesthesia workspace that can help to generate substantial reductions in SSIs. Post-induction wiping of the anesthesia machine and equipment, and two minutes of triangular UV-C treatment for augmentation of surface disinfection cleaning procedures are recommended for the anesthesia machine. In this study, we show that employment of these cleaning procedures by previously untrained anesthesiologists is both practical and safe. Based on these study results, post-induction wiping should be used to increase the frequency and quality of anesthesia machine and equipment cleaning following induction of anesthesia and patient stabilization, and two minutes of triangular UV-C treatment should be used to augment terminal surface disinfection cleaning procedures. Future work should explore the integration of UV-C for the augmentation of routine, between-case cleaning.
